# The Diagnosis and Management of Immune Checkpoint Inhibitor Cardiovascular Toxicity: Myocarditis and Beyond

**DOI:** 10.3390/vaccines10020304

**Published:** 2022-02-16

**Authors:** Dan Gilon, Zaza Iakobishvili, David Leibowitz

**Affiliations:** 1 Cardio-Oncology, Heart Institute, Hadassah Medical Center, Faculty of Medicine, Hebrew University of Jerusalem, Jerusalem 9112001, Israel; oleibo@hadassah.org.il; 2 Cardio-Oncology, Cardiology Department, Shamir Governmental Medical Center (Assaf Harofeh), Rishon Lezion 7528809, Israel; 3 Cardio-Oncology Service, Maccabi Health Services, Tel Aviv 6812509, Israel; 4 Department of Cardiology, Clalit Health Services, Tel Aviv District, Holon 5840608, Israel; zaza.iakobishvili@gmail.com; 5 Department of Cardiology, Faculty of Health Sciences, Samson Assuta Ashdod University Hospital, Ben Gurion University in the Negev, Beersheva 8410501, Israel

**Keywords:** immune therapy, immune checkpoint inhibitors, cancer, cardiovascular toxicity, myocarditis

## Abstract

Recent years have brought major advancements in the use of immune therapy and specifically immune checkpoint inhibitors (ICIs) in cancer patients, with expanding indications for various malignancies resulting in the treatment of a large and increasing number of patients. While this therapy significantly improves outcomes in a variety of hematologic and solid tumors, the use of ICIs is associated with a substantial risk of immune-related adverse events. Cardiovascular toxicity, while not the most common side effect of ICIs, is associated with significant morbidity and mortality. It is therefore crucial for oncologists and cardiologists, as well as internists and emergency room physicians, to have a good understanding of this increasingly common clinical problem. In the present review, we discuss the cardiac aspects of ICI therapy with special emphasis on the clinical manifestations of their cardiovascular toxicity, diagnostic approaches, treatment and suggested surveillance.

## 1. Introduction

**Figure 1:**
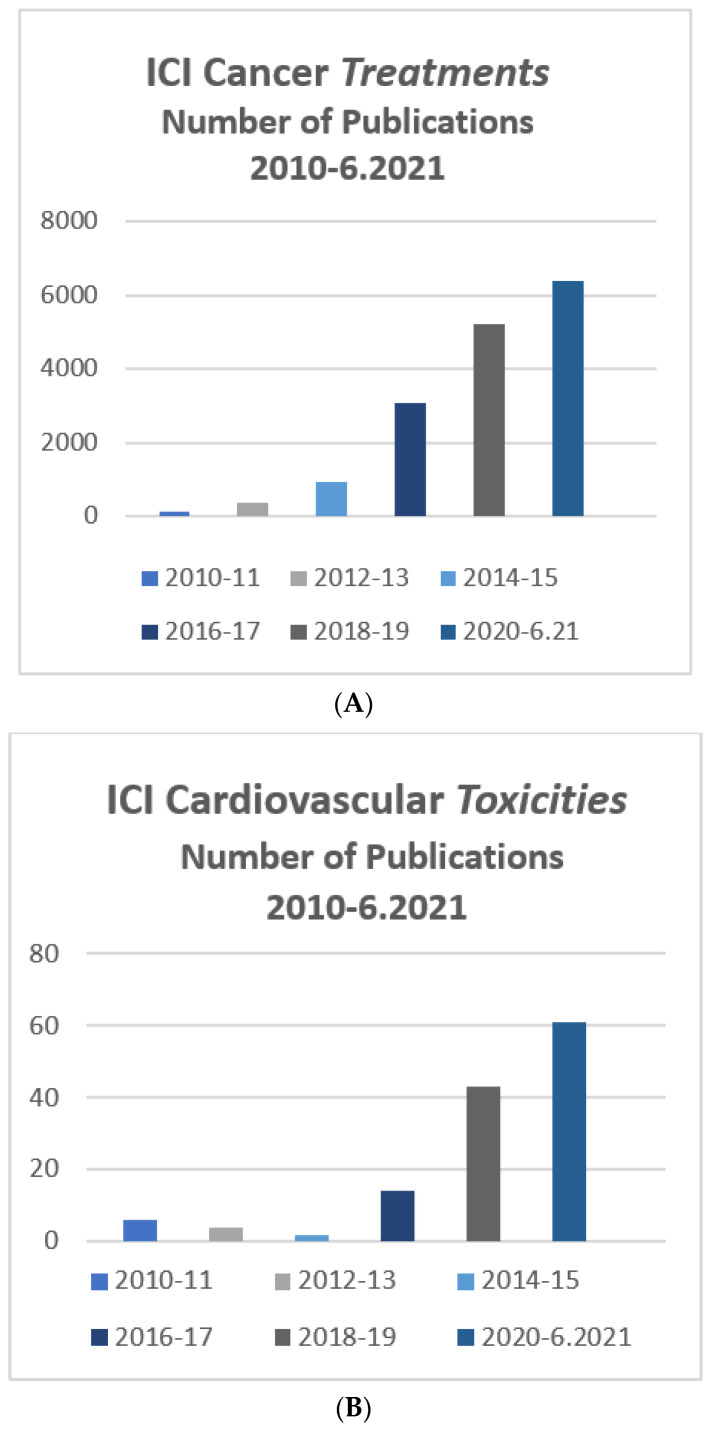
(**A**) Number of ICI treatment-related publications in recent years. (**B**) Number of ICI cardiovascular toxicities-related publications in recent years.

Recent years have brought major advancements in the use of immune therapy and specifically immune checkpoint inhibitors (ICIs) in cancer patients, with expanding indications for various malignancies resulting in the treatment of a large and increasing number of patients [1]. Malignant cancer cells are able to suppress the immune system to evade detection and destruction, in part due to the activation of inhibitor checkpoint pathways. ICIs are monoclonal antibodies that block this ability of cancer cells and permit an immune response against the tumor cells. The Food and Drug Administration approval for ICI therapy was first granted in 2011 and, to date, more than 50 indications have been approved. Importantly, this therapy is now being utilized earlier in the disease course of a variety of tumors. While this therapy significantly improves outcomes in a variety of hematologic and solid tumors, the use of ICI is associated with a substantial risk of immune-related adverse events (irAEs). Cardiovascular toxicity and, in particular, myocarditis, while not the most common side effect of ICIs, is associated with significant morbidity and mortality. The expanding clinical importance of this therapy is reflected in the growing number of publications in the medical literature relating to both ICI treatment and its important cardiovascular toxicities (Figure 1A,B). However, there is limited prospective data available to guide clinical decision-making with regard to the diagnosis and treatment of ICI-related cardiovascular toxicity.

**Figure 2:**
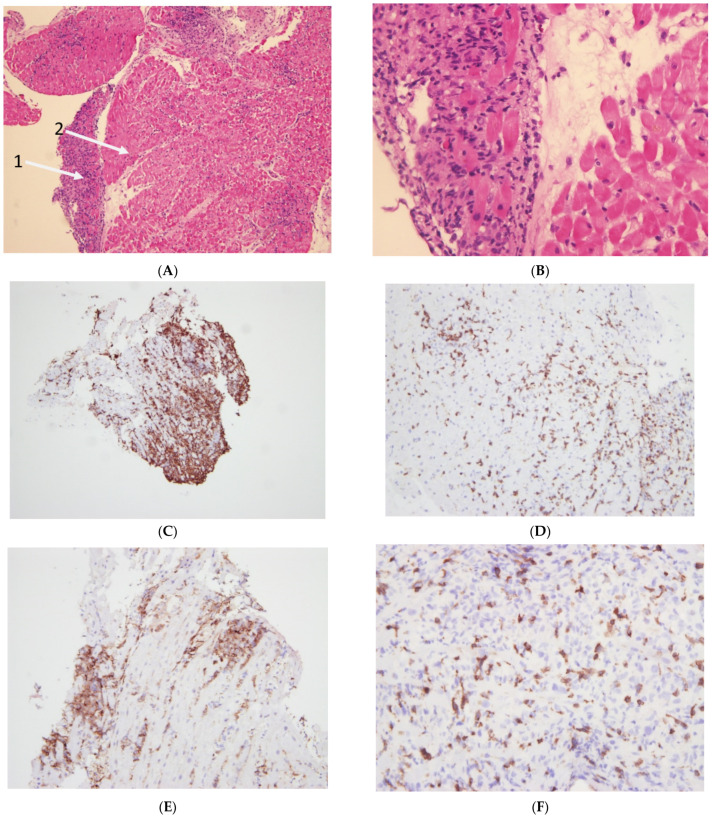

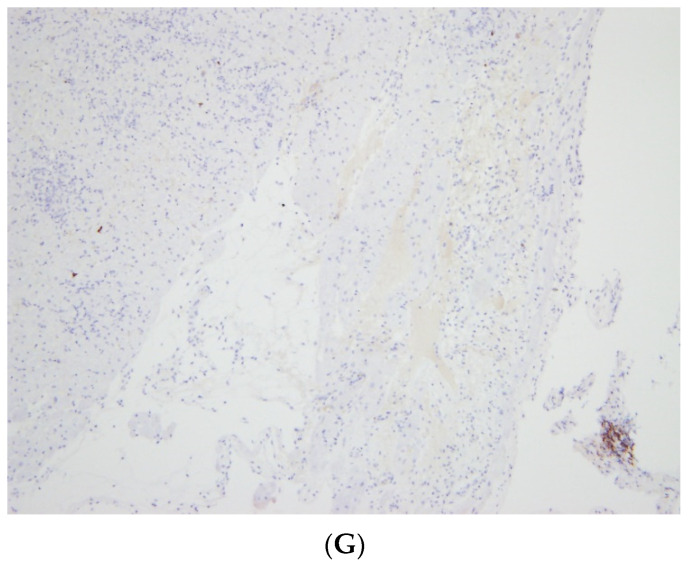
(**A**) Arrow 1—Dense infiltrate of inflammatory cells, mainly lymphocytes and neutrophils, with evidence of necrosis (on the left). Arrow 2—A region with edema but many more normal nuclei and without an infiltration of lymphocytes and neutrophils. (**B**) Enlarged image of Figure 1: Left: Significant infiltration of inflammatory cells with necrosis and loss of normal myocardial tissue. Right: Near normal tissue with mild edema. (**C**) CD163 staining for macrophages with a very intense and large area positive for macrophages. (**D**) CD3 staining of lymphocytes. (**E**) CD4 staining representing a subpopulation of lymphocytes. (**F**) Staining for CD8 representing another subpopulation of lymphocytes. (**G**) Cells with CD20 staining demonstrating the near complete absence of B cells.

**Case Vignette:** A 55 year old female with a history of diabetes and a diagnosis of metastatic melanoma began therapy with a programmed cell death ligand 1(PD-L1) inhibitor. A routine troponin exam showed an elevated troponin level of 1194 ng/L. The patient was asymptomatic and an echocardiogram revealed a normal ejection fraction (EF) of 63%. The patient was admitted to the cardiology service for observation and treatment with ICIs was stopped. Over the next few days, the patient’s troponin levels continued to rise. Therapy with intravenous solumedrol of 1 g a day was begun, but the troponin continued to rise and reached 12,661 ng/L. Repeat echo showed a significantly reduced EF of 35%. A coronary angiography showed normal coronary arteries and an endomyocardial biopsy revealed acute and chronic inflammatory infiltrates (see Figure 2A–G). The patient remained clinically stable, with a gradual reduction in troponin levels and an improvement of EF on the echocardiogram. The patient was discharged on prednisone with an EF that returned to 55%.

In this review, we discuss the cardiac aspects of ICI therapy with special emphasis on the manifestations of their cardiovascular toxicity, diagnostic approaches, treatment and suggested surveillance [2].

ICIs have been demonstrated to improve progression free and overall survival in a variety of cancers such as melanoma, renal cell carcinoma, non-small cell lung cancer, refractory Hodgkin’s lymphoma and others [3,4]. The enhancement of immune responses by ICIs causes the systemic activation of T-cell responses producing a range of auto immune toxicities, generally occurring in the early phase of therapy. In general, irAEs are the result of several postulated mechanisms, including the deregulation of previously tolerated self-reactive T cells, as well as the cross-reactivity between the cancerous cells and normal tissues. Humoral immunity may be modulated by ICI therapy, and cytokine activation as a result of ICI therapy has been reported in the literature [5]. While ICI cardiovascular (CV) toxicity is related presumably to the clonal expansion of T lymphocytes, which act against antigens shared by tumor cells and cardiovascular tissues, the specific mechanisms remain unclear. Programmed cell death protein-1 ligand (PD-L1) appears to be upregulated in models of T-cell-mediated myocarditis in mice. Animal models deficient in cytotoxic t lymphocyte-associated protein-4 (CTLA-4) and programmed cell death protein-1 (PD-1), show T lymphocyte infiltration and severe myocarditis with a rise in cardiac biomarkers, such as troponin and cardiomyocyte necrosis, on pathology. In some of these models, the animals developed severe heart failure and sudden death [6]. T-cell lymphocytes appear to have a predilection for myocardial infiltration and a pathological examination of these models demonstrates T lymphocytes in the myocardium. This finding is consistent with the pathological findings in patients with ICI-related myocarditis, which demonstrated T-cell lymphocytic infiltration in the myocytes and the conduction system [7]. In one of the case series, high levels of muscle-specific antigens in tumor cells were found in 2 patients who developed myocarditis, supporting the idea that toxicity is related to the T-cell targeting of shared antigens (Figure 2A–G).

In addition to T-cell activation, other mechanisms may play a role in ICI CV toxicity. PD-1 knockout mice who developed autoimmune myocarditis demonstrated high titers of antibodies to cardiac troponin [8]. It should be noted that no evidence of B cell or antibody–antigen deposition in the myocardium has been found in humans receiving ICI therapy. Cardiomyocyte PD-L1 expression is upregulated in cardiac disease and PD-L1 signaling may have cardioprotective actions in this setting. Therefore, the inhibition of this pathway may cause non inflammatory myocyte dysfunction in hearts with preexisting disease, such as left ventricular hypertrophy.

T-cell-mediated responses may contribute to the progression of acquired heart disease [9]. In addition to the direct effects on lymphocytes, a general increase in inflammation observed in patients receiving ICIs may worsen the clinical manifestations of congestive heart failure (CHF). Systemic inflammation may contribute to plaque rupture in susceptible patients and lead to acute coronary syndromes. It has also been shown that defects in PDL-1 function in dendritic cells located in vessel walls can contribute to inflammatory vasculitis [10].

Another potential mechanism of ICI-related cardiotoxicity may be the interactions of these agents with previous cancer therapy. Previous treatment with cardiotoxic agents, such as anthracyclines, may lead to the exposure of myocardial antigens and potentiate ICI toxicity. This synergistic mechanism was demonstrated with radiation therapy as well [11].

## 2. Epidemiology of ICI Cardiotoxicity

Overall, ICI-related immune-related adverse events (irAEs) are common and include many systems. The incidence of cardiovascular irAEs is relatively low, but the fatality rate may be high, particularly in cases of ICI-associated myocarditis.

In a recently published single center retrospective study of a large cohort of 102,701 patients with a diagnosis of malignancy, 424 patients received at least one ICI. Sixty-two (14.6%) patients were diagnosed with at least one new cardiovascular disease after the initiation of ICI therapy. Of those, 5.6% developed heart failure. When two ICIs were administered sequentially, 6.1% developed heart failure and/or cardiomyopathy. Cardiovascular disease was diagnosed within a median of 63 days after the initiation of ICI treatment. Mortality in ICI-treated patients with a concomitant diagnosis of incident cardiovascular disease was higher, compared to those without (66% vs. 41.4%, odds ratio of 2.77) [12].

In a nationwide study in Denmark, D’Souza et al. examined the data of 25,573 patients with lung cancer and 13,568 with melanoma [13]. The hazard ratios for cardiac events were significantly increased in both groups for patients receiving anti PD-L1 or CTLA-4 treatment. The absolute one-year risk for peri/myocarditis was 1.8%.

In the study of Mahmood et al., the prevalence of myocarditis was relatively low at 1.14%, among the lowest of irAEs, but was associated with a high fatality rate of 48%. Other studies confirmed a high mortality rate ranging from 17–50% [14]. There was a median time of onset of 34 days after starting ICI (interquartile range: 21 to 75 days).

Given the growing number of ICI-treated cancer patients, more patients developing cardiovascular toxicities are presenting to emergency departments. There is therefore a specific need for rapid diagnosis and treatment also in this clinical setting. Yeung et al. address this relatively large sub-group of irAEs, including cardiac disease, presenting to the emergency department [15].

## 3. Clinical Manifestations of ICI Cardiotoxicity

### 3.1. Myocarditis

#### 3.1.1. Clinical Presentation

As with many clinical conditions, the basis of the diagnosis of ICI-related myocarditis is a high level of clinical suspicion. The clinical presentation of myocarditis in the setting of ICI therapy is broad and may include chest pain with or without symptoms related to ventricular dysfunction, such as shortness of breath and fatigue. A relatively high proportion of patients with ICI myocarditis also develop symptoms of myositis, and up to 10% of patients may develop myasthenia gravis [16]. The clinical presentation may be fulminant with hemodynamic instability, life-threatening arrhythmias and multiorgan failure or more indolent. ICI myocarditis may also be asymptomatic, with only the elevation of biomarkers, such as troponin, as is exemplified in the case presented. Other clinical syndromes that may present with complaints, such as chest pain, or discomfort, such as acute coronary syndrome or pulmonary embolism, need to be excluded. Clinical risk factors, which should raise the suspicion of myocarditis, have been described and include combination ICI therapy, age, female sex, diabetes mellitus and elevated body mass index [17]. Zamani et al., utilizing the Food and Drug Administration reporting system, showed that myocarditis was more common with combination ICI therapy with an odds ratio of 1.93 [18]. A large retrospective pharmacovigilance database confirmed the finding that combination ICI therapy was significantly associated with fatal myocarditis, and clinicians should be particularly suspicious of this diagnosis in these patients [19].

When ICI myocarditis is clinically suspected, initial diagnostic testing should include electrocardiogram (ECG), biomarkers and echocardiogram.

#### 3.1.2. Electrocardiogram

ECG is an easily performed bedside test and, reportedly, is abnormal in up to 89% of patients with ICI myocarditis. The abnormalities may be subtle, such as tachycardia, mild QT prolongation and non-specific ST segment or T wave changes, and a normal ECG does not rule out the diagnosis of myocarditis.

Zlotoff et al. examined the ECG from a registry of myocarditis in ICI patients [20]. The study included 140 cases of myocarditis that were compared to 179 of the control cases. The QRS, but not the PR or QT interval, was found to be prolonged with myocarditis. A prolonged QRS duration was associated with an increase in subsequent MACE (HR 3.26). Each 10 millisecond increase in the QRS duration conferred a 1- to 3-fold increase in the odds of MACE.

#### 3.1.3. Biomarkers

As in the cases of suspected non-ICI-related myocarditis, levels of cardiac biomarkers, including troponin and brain natriuretic peptide (BNP) levels, should be measured. In one series, more than 90% of patients had elevated troponin while over 60% had elevated BNP [12]. Patients with major adverse cardiac events (MACEs) had significantly higher troponin levels than those without, demonstrating the prognostic importance of biomarkers in this setting.

### 3.2. Imaging

The most common modalities include echocardiography and cardiac magnetic resonance (CMR).

#### 3.2.1. Echocardiography

Given its ease and availability, echocardiography should generally be the initial imaging performed. Findings may include global or segmental ventricular dysfunction as well as pericardial effusion (observed in 7–17% of patients). However, it is important to note that close to 50% of patients with documented ICI myocarditis had a left ventricular ejection fraction (LVEF) in the normal range, so a normal EF on echocardiography does not rule out the diagnosis. On the other hand, a reduction in EF may be profound with 46% of patients having an LVEF < 35%, as reported in the study of Escudier et al. [21]. Standard 2 dimensional imaging may lack sensitivity for identifying ventricular dysfunction, and strain imaging should be performed as well. In a recent retrospective study, Awadalla et al. demonstrated a reduction in the global longitudinal strain (GLS) in patients with suspected ICI myocarditis and preserved LVEF [22]. In contrast, patients receiving ICI without clinical myocarditis had preserved GLS. Reduced GLS (<16%) was associated with increased MACE, particularly in patients with preserved EF (>50%). Therefore, an assessment of patients with ICI myocarditis with GLS appears to be important for appropriate risk stratification, however future prospective studies are necessary to confirm the role of GLS in these patients [22].

#### 3.2.2. Cardiac Magnetic Resonance (CMR)

Cardiac magnetic resonance imaging (CMR) is considered the gold standard imaging modality in the diagnosis of myocarditis, due to its excellent spatial resolution and ability to provide tissue characterization. Criteria have been established for the diagnosis of myocarditis using CMR. This technique is expensive, not available in all centers and may be difficult to perform in critically ill patients. Until recently, limited data were available on the use of CMR in the setting of ICI myocarditis. In a registry study, Zhang et al. reported CMR findings in 103 patients with ICI myocarditis [23]. Similar to the echocardiographic findings, 61% of patients had an EF ≥50%. Late gadolinium enhancement (LGE) was observed in 48%, but did not correlate with reduced EF and was not associated with MACE. In contrast, LGE was present in >80% of patients with non-ICI myocarditis. The authors noted time dependency in the presence of LGE, with the increased diagnosis of LGE on exams performed at least 4 days after admission. These findings suggest that the sensitivity of LGE on CMR for the diagnosis of ICI myocarditis may be limited. A recent study examined the use of T1 and T2 mapping in this clinical setting. Abnormal T1 and T2 values were seen in 78% and 43% of the patients, respectively. Native T1, but not T2 levels, were associated with subsequent MACE. Therefore, the use of T1 mapping appears mandatory when utilizing MRI to assess ICI-related myocarditis [24].

#### 3.2.3. Endomyocardial Biopsy (EMB)

EMB is the gold standard test for the diagnosis of myocarditis. It should generally be performed by experienced operators in specialized centers. Even in experienced hands, sensitivity is about 70%, depending on the number of samples taken [25]. There are limited data on the sensitivity of EMB in the setting of ICI myocarditis. Previous pathological reports described lymphocytic infiltration in a majority of patients with suspected ICI myocarditis undergoing EMB with immunohistochemical staining showing predominately CD8 + T lymphocytes. Rarely, EMB may demonstrate other etiologies of myocarditis in ICI-treated patients. Given its invasive nature and limited sensitivity, EMB should probably be reserved for unstable, rapidly deteriorating patients or patients in which other non-ICI related etiologies are suspected [26,27].

## 4. Non-Myocarditis ICI-Related Cardiotoxicity

**Takotsubo syndrome:** Takutsubo syndrome (TTS) is a syndrome of acute, transient regional, mostly apical, left ventricular dysfunction in the absence of obstructive coronary artery disease. The clinical presentation shares many features of myocardial infarction, such as acute chest pain, ECG changes, including ST elevations, and elevated troponin. Various sets of diagnostic criteria have been proposed. Its pathophysiology remains uncertain and recent advances in the field have emphasized the importance of catecholamine-induced myocardial stunning triggered by physical or emotional stress [28,29].

Several authors have reported patients with clinical evidence of TTS following treatment with ICI. Serzan et al. presented a case of a patient treated with the (anti-CTLA-4) agent ipilimumab and the anti-PD-1, nivolumab who presented with dyspnea on exertion and generalized pain. The ECG showed a new inferolateral T wave inversion suggesting ischemia [30]. TTE showed classical apical akinesis with hyperdynamic basal LV segments and the high-sensitivity cardiac troponin I level was elevated. Coronary catheterization revealed nonobstructive coronary artery atherosclerosis. These results, along with cardiac magnetic resonance imaging, were suggestive of Takotsubo cardiomyopathy. ICI-related myocarditis could not be excluded and an endomyocardial biopsy was obtained. Pathology results did not meet the WHO criteria for a pathological diagnosis of myocarditis. This combination of clinical presentation, laboratory findings and imaging and especially the use of endomyocardial biopsy enabled the diagnosis of Takotsubo cardiomyopathy and avoided unnecessary immunosuppressive therapy.

This report focused on the role of endomyocardial biopsy, not only to diagnose ICI-related myocarditis, but also its importance in ruling out myocarditis in borderline cases. Previously described variants of TTS, including a “reverse” or “inverted” pattern of basilar hypokinesis, were described in patients receiving ICI as well. While the true incidence of TTS following treatment with ICI remains unclear, clinicians caring for these patients should be aware of this potential clinical diagnosis [31].

**Pericardial disease:** Pericardial disease is a relatively common cardiac complication of ICI therapy. In an important retrospective study at a single academic center, Gong et al. compared 2842 consecutive patients who received ICIs with 2699 age- and cancer-type matched patients with metastatic disease who did not receive ICI [32]. A pericardial event was defined as a composite outcome of pericarditis and new or worsening moderate or large pericardial effusion. The endpoints were obtained through a chart review and were blindly adjudicated. There were 42 pericardial events in the patients treated with ICI (n = 2842) over 193 days (IQR: 64–411), yielding an incidence rate of 1.57 events per 100 person years. There was a more than four-fold increase in the risk of pericarditis or a pericardial effusion among patients on an ICI, compared with controls not treated with ICI after adjusting for potential confounders (HR 4.37, 95% CI 2.09 to 9.14). The conclusions of the study were that ICI use was associated with an increased risk of the development of pericardial disease among patients with cancer, and that a pericardial event on an ICI treatment was associated with a trend towards increased mortality.

**Arrhythmia**: Arrhythmias secondary to ICI therapy include ventricular arrythmia, conduction abnormalities and atrial fibrillation. When considering the incidence of arrhythmias in ICI-treated patients, it is important to note that in most series and reviews there is no clear distinction between the arrhythmias being associated with myocarditis or other irAEs.

In a retrospective study, Salem et al. completed a disproportionality analysis based on adverse drug reactions reported within VigiBase (a database managed by Uppsala Monitoring Center) containing more than 16 million individual case safety reports (ICSRs) submitted by national pharmacovigilance centers since 1967. They reported that supraventricular arrhythmias in ICI-treated patients (n = 222) were overwhelmingly associated with other concurrent irAEs, such as gastrointestinal disorders (93 (41.9%) of 222; mainly colitis and diarrhea leading to dehydration and electrolyte disorders), other cardiac conditions (69 (31.1%) of 222, mainly cardiac dysfunction, cardiac ischemia and pericardial disorders), endocrine disorders (66 (29.7%) of 222; mainly thyroid abnormalities), and neurologic disorders (28 (12.6%) of 222, including strokes and encephalitis) [33]. Escudier et al. analyzed 30 patients from 2 cardio-oncology units with ICI-related cardiotoxicity. They reported that atrial fibrillation was observed in 30% of the patients, ventricular arrhythmia in 27%, and conduction disorders in 17%. The arrhythmias were isolated (without left ventricular systolic dysfunction) in 3%, 7%, and 13% of patients, respectively, suggesting that arrhythmias can be a manifestation of ICI cardiac toxicity independently of associated myocarditis [21]. It should be taken into account, when considering the incidence of atrial fibrillation occurrence in ICI-treated patients that in most series there is no clear distinction between the atrial fibrillation as being myocarditis-associated, as non-myocarditis ICI cardiac toxicity, or secondary to other irAEs, as mentioned earlier.

**Vascular complications:** In a retrospective study, Bar et al. analyzed the incidence of acute vascular complications among 1215 patients with cancer who received ICI therapy. Approximately 1% of the patients developed a myocardial infarction or ischemic stroke within 6 months after the initiation of ICI treatment [34]. Additionally, a recently published systematic review analyzed the incidence of arterial thrombotic events, in particular, stroke and myocardial infarction, following ICI therapy [35]. Among the 17 studies evaluated, with a total of 10,106 subjects, the incidence rate of arterial thrombotic events in ICI-treated patients was 1.1%. The onset was relatively delayed with a median of 55 days (2–98 days). Drobni et al. demonstrated a three-fold higher risk of cardiovascular events at 2 years and an increase in cardiovascular events from 1.37 to 6.55 per 100 person years (adjusted hazard ratio 4.8) in ICI-treated patients [36]. Simultaneously, they conducted an imaging sub-study in patients with melanoma who were treated with an ICI. They measured the thoracic atherosclerotic plaque burden over time. The rate of progression of the total aortic plaque volume was more than 3-fold higher with ICI treatment. They also showed that both were attenuated with concomitant use of statins or corticosteroids [37]. Given the relationship between atherosclerosis and inflammation, as well as evidence that ICIs aggravate existing inflammatory diseases, Poels et al. studied the propensity of short-term ICI therapy to aggravate atherosclerosis [38]. They used 18F-FDG (2-deoxy-2-[fluorine-18] fluoro-D-glucose) positron emission tomography-computed tomography to detect macrophage-driven vascular and systemic inflammation in pembrolizumab and nivolumab/ipilimumab-treated melanoma patients. They also treated atherosclerotic mice with CTLA-4 and PD-1 inhibition to study the proinflammatory consequences of immune checkpoint inhibition. Their study demonstrated that combination therapy with anti-CTLA-4 and anti-PD-1 antibodies does not affect myeloid-driven vascular and systemic inflammation in melanoma patients and hyperlipidemic mice. However, short-term ICI therapy in mice induced T-cell-mediated plaque inflammation and drove plaque progression. The authors suggest, based on their study and others, that additional risk stratification strategies may be required to identify the individuals who are at risk of developing cardiovascular disease following ICI therapy.

**Vasculitis** is a known irAE of ICI treatment, generally clinically less severe than others. Salem et al. reported, based on their observational study, that the most common vasculitis syndromes were polymyalgia rheumatica and temporal arteritis [33].

## 5. Treatment of ICI-Associated Myocarditis

It is important to note that no randomized data are available and that treatment recommendations are based on case series and expert opinion. There is general agreement that withholding ICI treatment is the critical first step once ICI-related myocarditis has been diagnosed. Generally high dose corticosteroids are regarded as the first stage of immunosuppressive therapy [39,40]. Their role in the treatment response is based on their effect on the large exaggerated T-cell overactivation caused by the immune therapy [41]. IV Methylprednisolone of 1000 milligram (mg)/day is recommended as a pulse dose usually for three days, followed by 1 mg/kg daily either intravenously or orally. The American Society of Clinical Oncology guidelines recommend a tapering of at least 4–6 weeks. However, specific tapering should be tailored on a case-by-case basis. Zhang et al. found that time initiation of steroid treatment impacted MACE-free survival, whereby patients receiving corticosteroids within 24 h, regardless of dosage, showed the best outcome, and patients receiving corticosteroids after 72 h, regardless of dosage, showed the worst outcome [42]. A similar treatment approach was recommended in a recent review by the French Working Group’s plea for a pragmatic approach [43].

Generally, after several days of high dose intravenous therapy, oral steroid therapy replaces IV therapy, and is gradually tapered over 4–6 weeks. The optimal length of immunosuppressive treatment remains unclear but should presumably be continued until the symptoms have resolved, and biomarker levels as well as LVEF have returned to normal. There are sporadic reports in the literature of treatment with several agents, such as intravenous immunoglobulins, anti-thymocyte globulin, mycophenolate and abatacept, in patients who remain unstable or who fail to respond adequately to corticosteroids, for which the length of treatment is even less clear [44,45].

In addition to immunosuppressive therapy, conventional cardiovascular therapy including renin-angiotensin inhibition, beta blockers and diuretics should be provided when clinically necessary. Patients with elevated biomarkers or reduced LVEF on imaging should be managed in a monitored cardiology ward. Patients with hemodynamic instability should be managed with inotropic therapy and advanced mechanical support as needed. Other manifestations of ICI cardiotoxicity, such as pericarditis or arrhythmias, should be managed with conventional treatment.

## 6. Surveillance

All patients receiving ICI therapy should have a baseline evaluation prior to ICI treatment initiation, including medical history, a physical examination along with ECG and troponin levels. Echocardiograms are also performed in many centers. The follow-up and surveillance approach should be implemented on a routine basis and adapted to the specific patients as necessary.

There is no current consensus on how to screen for ICI cardiotoxicity. The proposed strategies have mainly included measurement of biomarkers, such as troponin and/or BNP, but many centers do not routinely screen. The use of echocardiography for surveillance is limited by the fact that, as noted earlier, many patients may have myocarditis with normal or near normal EF. In a protocol described by the Stanford group, they reported the experience with 214 patients receiving either monotherapy or combination ICI. Following baseline evaluation, patients were followed for 9 months using high sensitive troponin I (hsTnI) as their surveillance biomarker upon every treatment (up to 10 cycles at intervals of 2–4 weeks). When troponin was elevated, a more extended evaluation was performed by a multidisciplinary cardio-oncology team. During follow-up, out of the 214 patients, 24 had hsTnI over 55 ng/L. Of these, 3 patients were diagnosed as having ICI-related myocarditis, while the 21 other patients with elevated hsTnI were diagnosed with myocardial injury of various etiologies. Further studies are necessary to validate this approach to surveillance [46].

## 7. Rechallenge of ICI Therapy after Myocarditis

An interesting and unresolved question is that of re-instating ICI therapy after recovery from myocarditis. Several studies have reported successful re-institution of ICI therapy after recovery from myocarditis, however there have been reports of fatal recurrence of myocarditis as well, after re-instating the ICI therapy. Evidently, the re-institution of ICI therapy should be avoided in patients who developed hemodynamic instability or complications, such as arrhythmias, as well as those whose ventricular function remained reduced. In mild cases of ICI cardiac toxicity, particularly in those patients without other cancer-related therapeutic options, re-instating ICI therapy could be considered after review by the cardio oncology team. Such patients would require careful clinical surveillance utilizing biomarkers and echocardiography.

Dolladile et al. looked into irAEs in an observational, cross sectional, pharmacovigilance cohort study that examined case safety reports from VigiBase, which obtained case reports from more than 130 countries [47]. In this cohort study of 24,079 immune-related adverse events associated with at least one ICI, the recurrence rate of the same immune-related adverse event that prompt discontinuation of ICI therapy was 28.8% after patients received a rechallenge with the same ICI. A different irAE occurred in 4.4% of patients. In a rechallenge, colitis, hepatitis and pneumonitis had a higher recurrence rate compared with other immune-related adverse events. The overall results and the complexity involved in such cases once again emphasize the need to make decisions on an individual basis, by a multidisciplinary expert working group.

## 8. Open Questions and Future Directions

The rapidly evolving new ICI treatments for cancer, while highly effective in a variety of pathologies, raise growing concern regarding immune-related adverse effects (irAEs). These concerns are particularly relevant to the cardiovascular system, which is the main focus of this review.

### 8.1. Two of the Important Issues That Need Further Consideration

#### 8.1.1. Diagnosis and Monitoring by Imaging

Nishino et al. addressed the issue of imaging of cancer immunotherapy. Beyond discussing current imaging approaches, they described clinical-related radiographic manifestations of organ-specific irAEs, where imaging has a “critical role in diagnosis and monitoring” [48]. They emphasized that imaging techniques using novel radioactive tracers that target key molecules of immune-checkpoint pathways and cellular immune responses have been explored [49]. Some examples include engineering high affinity PD-1 variants for immune-PET imaging and a humanized antibody for imaging immune checkpoint ligand PD-L1 expression in tumors [50,51]. In principle, the toxicities can involve various organs, including cardiovascular, where imaging of this type may be of potential assistance in monitoring and diagnosis [52,53].

#### 8.1.2. Combination Therapy

Another important change in the treatment of tumors in recent years is the use of multiple ICI treatments, at times in combination with non-ICI treatment. The use of these combined therapies is expected to grow and diversify. The additive effects of combination therapy on cardiovascular toxicity remains unclear and requires further study. Arecent relevant study Tawbi et al. addressed this issue [54]. The authors compared the antitumor activity and safety in previously untreated melanoma or unresectable melanoma by a combination treatment with relatlimab, a lymphocyte activating gene-3–blocking antibody, and nivolumab, a PD-1–blocking antibody. They were able to show that this combination treatment provided a greater benefit with regard to progression-free survival than inhibition of PD-1 alone. Along with all irAEs, they also demonstrated that myocarditis occurred in 1.7% of the patients in the relatlimab–nivolumab group and in 0.6% of those in the nivolumab group. Also important was their finding that myocarditis events in the relatlimib-nivolumab group resolved completely. More studies are needed to clarify the incidence and prognosis of ICI-related myocarditis in this setting.

## 9. Take Home Messages: ICI Cardiovascular Toxicities

***Major (issues and concepts):***

A range of cardiovascular toxicities may develop, with myocarditis being the most serious and potentially fatal (~50%)

- High degree of suspicion for myocarditis is mandatory in ICI treated patients
- Combination ICI therapy is an important risk factor, beyond other known risk factors, for myocarditis

**Diagnosis is based on:**

- Usually on variable combination of: clinical signs and, physical examination, ECG,
- Echo (global function, segmental wall evaluation, and strain),
- Cardiac Magnetic Resonance (CMR) imaging.
- Endomyocardial Biopsy—considered gold standard

**Treatment Approach:**

- ICI discontinuation.
- Immunosuppressive treatment based on steroids
- Relevant pharmacological and/or mechanical cardiac support is needed if steroid treatment fails or is insufficient

***Minor, but relevant and important to remember: ***

- Global left ventricular function (LVEF) may be normal in up to ~50% of patients with proven myocarditis.

Therefore, the importance of segmental wall motion abnormalities, additional imaging with MRI and when indicated, biopsy.

- **No consensus** on screening, surveillance and prevention for ICI-associated myocarditis.
- When myositis is diagnosed or suspected, the possibility of myocarditis should be also fully evaluated, due to high likelihood of co-existence.

ICI treatment re-institution: overall is considered to be associated with elevated risk, but is not prohibitive (limited literature) 

## Data Availability

Not applicable.
